# Early and Noninvasive Bird Gender Identification by ATR-FTIR Spectra Coupled with a Randon Forest Algorithm

**DOI:** 10.1021/acsomega.5c07984

**Published:** 2025-10-13

**Authors:** Silvano Dias PereiraNaves, Victor Fidelis Fernandes, Matheus Cicero Ribeiro, Thiago França, Giorgio S. Senesi, Simone Sanches, Cleber Galvão, Cynthia Mantovani, Cícero Cena, Bruno Marangoni

**Affiliations:** † Programa de Pós-Graduação em Ciência dos Materiais, 54534 UFMS—Universidade Federal de Mato Grosso do Sul, Avenida Costa e Silva s/n, 79090-900 Campo Grande, Brazil; ‡ 9327 CNR—Istituto per la Scienza e Tecnologia dei Plasmi (ISTP) sede di Bari, Via Amendola 122/D, 70126 Bari, Italy; § Codex Gen—GSM Biologia Molecular e Biotecnologia, Avenida Tamandaré 6000, Bloco L - Biotecnologia, 79117-900 Campo Grande, Brazil

## Abstract

The early identification of sex in birds is essential for reproduction, breeding programs, and commercialization and plays a crucial role in wildlife management and environmental law enforcement. The DNA-based molecular techniques, known for their accuracy and noninvasive nature, are the primary methods for sex determination. However, these techniques are time-consuming and expensive and require specialized laboratories. This study explores the use of attenuated total reflectance Fourier transform infrared spectroscopy (ATR-FTIR) combined with the Random Forest algorithm as a noninvasive, cost-effective, and precise alternative for early gender determination in birds. Measurements were performed on the feather region, known as the vexillum, of four bird species: *Oryzoborus maximiliani* (Bicudo), *Nymphicus hollandicus* (Cockatiel), *Oryzoborus angolensis* (Curio), and *Psittacula krameri* (Ring-necked Parakeet). The bird’s sex was confirmed by DNA analysis. The ATR-FTIR spectra in the range of 3800–800 cm^–1^ were processed using standard normal variate (SNV) and analyzed with principal component analysis (PCA) to reduce dimensionality and highlight significant transitions. Processing ATR-FTIR spectra by the Random Forest classifier yielded promising results, with accuracy rates in an external validation of 94.4% for Bicudo and Curio, 77.8% for Cockatiel, and 72.2% for the Ring-necked Parakeet. These findings highlight the potential of ATR-FTIR as a viable technique for the early identification of gender in birds.

## Graphical abstract

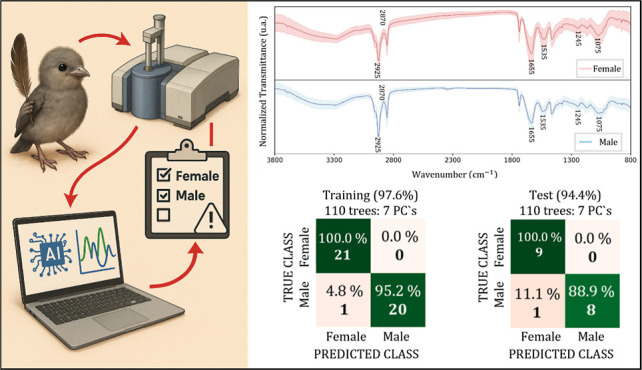

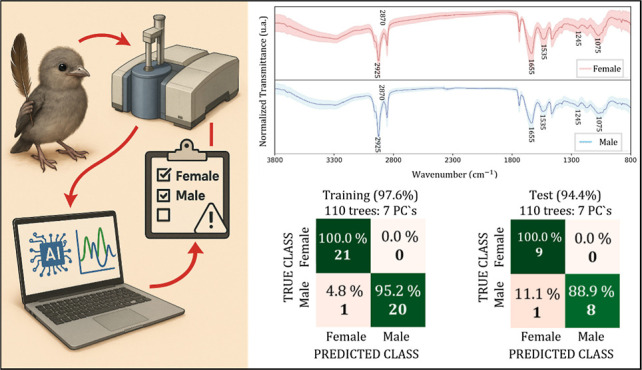

## 1 Introduction

The need for the early identification of gender in birds is widely recognized for its direct benefits to reproduction, breeding, and commercialization.^1^ From the wildlife management and environmental law enforcement aspects, this practice plays a fundamental role in crucial areas such as the preservation of endangered species, the study of sex-related diseases, genetic improvement, and other initiatives that require innovative solutions to meet the growing demands of the market.^1,2^

The standard procedure for bird gender determination is based on accurate, reliable, and risk-free DNA molecular analysis.^3^ However, it is costly, time-consuming, and often requires specialized laboratories.^3–5^ Other available methods, such as endoscopy, laparoscopy, cytogenetics, morphological analysis (shapes, colors, sizes), behavioral analysis, and sexual dimorphism, frequently face various limitations/constraints, including uncertainties or the need for sexual maturity.^6,7^

In the context of technological advancements and the emergence of automated techniques, new less costly methods for early bird sex have been developed and applied in the last years, including Fourier transform infrared spectroscopy (FTIR).^8–10^ In particular, FTIR spectroscopy was used to analyze growing contour feathers of turkey chicks, which showed spectral variations in the range of 1000–1250 cm^–1^. Classification using unsupervised principal component analysis (PCA) enabled the classification of chicks as male or female with over 95% accuracy.^8^

In another study,^9^ two DNA- and RNA-related FTIR absorption bands at 1080 cm^–1^ and at 1120 cm^–1^ were identified. In particular, the absorption band around 1120 cm^–1^ was attributed to the RNA ribose C–O stretching that is responsible for the male bird’s faster growing and later a stronger body. In a later study,^10^ the same authors demonstrated that only a small amount of the feather pulp was necessary to determine the sex of pigeons. FTIR spectra processed with PCA and classified using Linear Discriminant Analysis (LDA) provided sex-related information through principal components (PC) 2 and 4. In particular, PC2 reflected varying protein amounts in the two genders, while PC4 revealed variations in the amide I and amide II bands and in the phosphate vibrations region of nucleic acids. As a result, male feather pulp samples were classified with 100% accuracy.

In the context of the rapid evolution of technology and the emergence of Artificial Intelligence (AI) as a knowledge-based, intelligent, and continuously learning system, companies are relentlessly seeking improvements in automation, efficiency, and better data comprehension, leading to increasingly complex decision-making processes.^11,12^

In this study, we apply attenuated total reflectance (ATR)-FTIR combined with a Random Forest algorithm to the feathers of the birds *Oryzoborus maximiliani* (Bicudo), *Nymphicus hollandicus* (Cockatiel), *Oryzoborus angolensis* (Curio), and *Psittacula krameri* (Ring-necked Parakeet) to develop a protocol able to distinguish male and female birds within each species. The FTIR molecular vibrational transitions are discussed, and the limitations and promising potential of the technique are highlighted.

## 2 Materials and Methods

### 2.1 Sample Collection and Preparation

The analyzed samples were provided by “Codex Gen”, a bird sexing company in Campo Grande, MS, and consisted of feathers from the adult male and female birds of the species *O. maximiliani* (Bicudo), *N. hollandicus* (Cockatiel), *O. angolensis* (Curio), and *P. krameri* (Ring-necked Parakeet). Samples were collected from 30 animals per sex, with two measurements taken from each animal and subsequently averaged. This procedure yielded 60 samples per species, resulting in a total of 240 samples in the data set. All samples were previously identified by DNA analysis.

### 2.2 FTIR Setup

The FTIR spectra were acquired in the mid-infrared (MIR) region from 3800 to 800 cm^–1^, with a resolution of 4 cm^–1^, and 12 scans, using a PerkinElmer Spectrum 100 Series spectrometer. The ATR accessory used was provided with a ZnSe crystal.

### 2.3 Data Analysis

The spectra were first visually analyzed to identify any possible error that occurred during their measurement. Then, an average spectrum was obtained for each duplicate. The spectra were preprocessed and normalized using the Standard Normal Variate (SNV) method, which sets each spectrum mean to 0 (zero) and the standard deviation to 1 (one), so reducing the offset and standardizing the intensities,^13^ according to

1 $$x_{ij}=\frac{x_{ij}-\mu_{i}}{\sigma_{i}}$$

where *x*_*ij*_ is the transmittance intensity *j* of the spectrum *i*, and μ_*i*_ and σ_*i*_ are, respectively, the spectrum mean and standard deviation.

To perform the spectral exploratory analysis, the principal component analysis (PCA) method was applied to the FTIR spectra with the aim of reducing the dimensionality and seeking clusterization/differentiation trends. Three different spectral ranges were investigated, i.e., 3800–800 cm^–1^, 3000–2800 cm^–1^, and 1800–800 cm^–1^. The PCA was performed by the eigenvalue and eigenvector decomposition of the sample data set, so achieving a new set of linearly uncorrelated variables called principal components (PCs). The projection of the spectra onto these PCs are called “scores”, the scatter of which shows the trends of similarities or differences among the samples. The PCs are sorted in the descending order of explained variance (%), i.e., the first PC involves the highest variance, the second PC the second highest variance, and so on.^14^

The Random Forest is a supervised machine learning algorithm used for classification and regression, consisting of an ensemble method that builds multiple decision trees during training and combines their outputs to improve prediction accuracy and reduce overfitting. Each tree is trained on a random subset of the data by means of the bootstrapping method for samples and variables (features). The final predictions are aggregated through majority voting (for classification) or averaging (for regression).^15,16^

In classification tasks, a confusion matrix is usually achieved to evaluate the model’s performance. The confusion matrix is a table that compares the predicted and actual class labels, providing insights into the model capability to correctly predict the true class of the out-of-training samples set. For a binary classification task, the two different sample classes are set as positive and negative classes. The metrics obtained are related to the quantity of samples predicted by the model and are described as true positives (TP), i.e., correctly predicted positive samples, true negatives (TN), i.e., correctly predicted negative samples.^17^ The overall model accuracy is then calculated according to the following formula

2 $$accuracy\;\left(\%\right)=\frac{\left(TP+TN\right)}{\left(total\;samples\right)}$$

## 3 Results and Discussion

### 3.1 Spectral Exploratory Analysis

The average FTIR spectra for each bird species are shown in Figure 1. Given the spectral similarity between male and female species, the data were combined and averaged across all samples within each species, aiming at identifying the main absorption bands and attributing them to characteristic vibrational modes of specific chemical bonds. The observed bands (Table 1) are consistent with those reported in the literature for the bird feather vexillum.^18^

**Figure 1:**
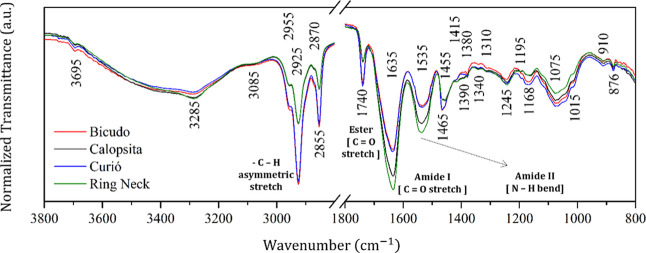
Average FTIR spectra of each bird species with the main wavenumbers and their corresponding biochemical assignments highlighted.

**Table 1:** Peak Assignment and Their Vibrational Modes in FTIR Spectra of Bird Feather Vexilla

wavenumber (cm^–1^)	assigned peak	vibrational modes
1075	C–C	symmetrical stretching
1168	C–C	symmetrical stretching
1245	(CN) amide III	symmetrical stretching
1310	C–H_2_	scissoring bending
1380	C–H_3_	scissoring bending
1436	C–H_3_	scissoring bending
1455	C–H_3_	scissoring bending
1535	(N–H) amide II, β-sheet	scissoring bending
1655	(C=O) amide I, α-helix	symmetrical stretching
1666	(C=O) amide I, β-sheet	symmetrical stretching
1680	C=O	symmetrical stretching
2870	CH_2_	symmetrical stretching
2925	CH_3_	symmetrical stretching
2955	CH_3_	asymmetrical stretching
3085	NH amide B	asymmetrical stretching
3285	NH amide A, α-helix	symmetrical stretching

Based on the exploratory analysis, a notable spectral similarity was observed between the Bicudo and Curio species as well as between the Cockatiel and Ring-necked Parakeet. However, distinct absorption bands were identified at approximately 1390, 1415, and 1195 cm^–1^, which may be attributed to species-specific biochemical characteristics, as well as to other factors related to feather collection and/or characterization.

Due to the spectral similarity among the bird species, we chose to perform sex determination separately for each species to avoid confusion during algorithm training, as species identification was not a primary objective of this study. For clarity and coherence in the presentation of the results, we first present the analysis for *O. maximiliani* (Bicudo) in the following section with a more detailed discussion. The results for the remaining three species, *O. angolensis* (Curio), *N. hollandicus* (Cockatiel), and *P. krameri* (Ring-necked Parakeet), are summarized in the subsequent section.

### 3.2 *Oryzoborus maximiliani* (Bicudo)

The average and standard deviation spectra of the Bicudo bird feather were quite similar for the two genders (female and male; Figure 2). Furthermore, the standard deviation of the female samples was higher than that of male samples, which may influence data variance during the exploratory PCA stage.

**Figure 2:**
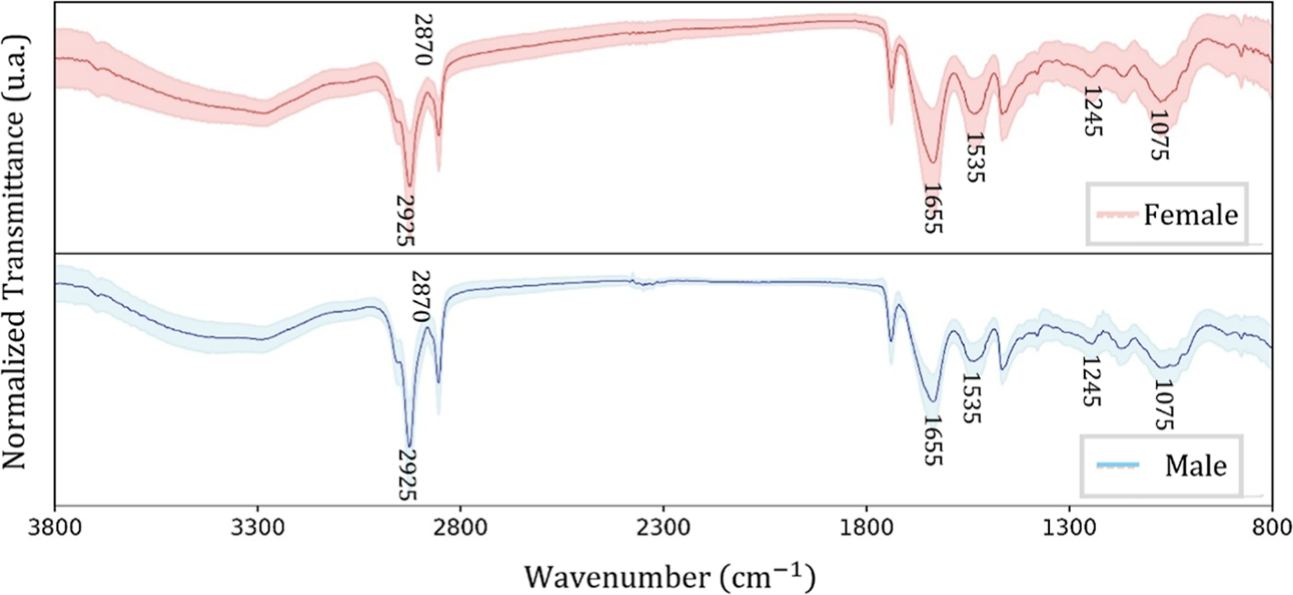
Average FTIR spectra (solid line) and their standard deviation (shaded area) for the Bicudo bird females (red) and males (blue).

Before applying PCA to the 60 samples of each species, i.e., 30 male and 30 female samples, the samples were randomly divided into two sets: a training set (70% of sample) and a test set (remaining 30%). Then, the scores obtained for each range and each training set were used as inputs to train the Random Forest classifier, optimizing the model through Leave-One-Out Cross-Validation (LOOCV).

The score plot and loading plot achieved by PCA applied to the spectral range from 3800 to 800 cm^–1^ for the Bicudo bird are shown in Figure 3. In particular, the score plot showed a relevant dispersion in the data for the female class compared with the male class, for which a trend of clustering formation is evident. The loadings plot indicated that the main spectral contributions were in the regions 3000–2800 cm^–1^ and 1800–1300 cm^–1^ (Figure 3), which correspond to characteristic vibrational modes of lipids and proteins.^19–21^ The first region (3000–2800 cm^–1^) comprises the C–H stretching vibrations of methylene and methyl groups (CH_2_ ∼ 2920 cm^–1^, CH_2_ ∼ 2850 cm^–1^, and CH_3_ ∼ 2956 cm^–1^), typically associated with the hydrocarbon chains of lipids, waxes, and fatty acids.^22^ These bands are strongly related to surface lipid layers in feathers, including wax esters secreted by the preen gland, which play a role in waterproofing, protection, and potentially intraspecific communication. The second region (1800–1300 cm^–1^) encompasses both lipid and protein contributions. A band around 1740 cm^–1^ is assigned to the ester carbonyl stretching of triglycerides and wax esters, reinforcing the lipid-related variability.^22^ In contrast, the amide I (∼1650 cm^–1^, C=O stretching) and amide II (∼1540 cm^–1^, N–H bending, and C–N stretching) bands originate from keratin, the main structural protein in feathers, and provide information on protein conformation and the secondary structure.^23^ The amide III region (1300–1220 cm^–1^) also contributes, reflecting C–N stretching and N–H bending modes in keratin, with a possible overlap from phosphate vibrations in phospholipids.^24^ The loadings suggest that the separation observed in the multivariate models arises primarily from variations in the lipid content and composition combined with subtle differences in the keratin structure. Biologically, these findings may reflect interspecific or sexual differences in the secretion and deposition of preen gland lipids as well as structural variations in the keratin matrix of feathers, both of which could be influenced by ecological, physiological, or reproductive factors.

**Figure 3:**
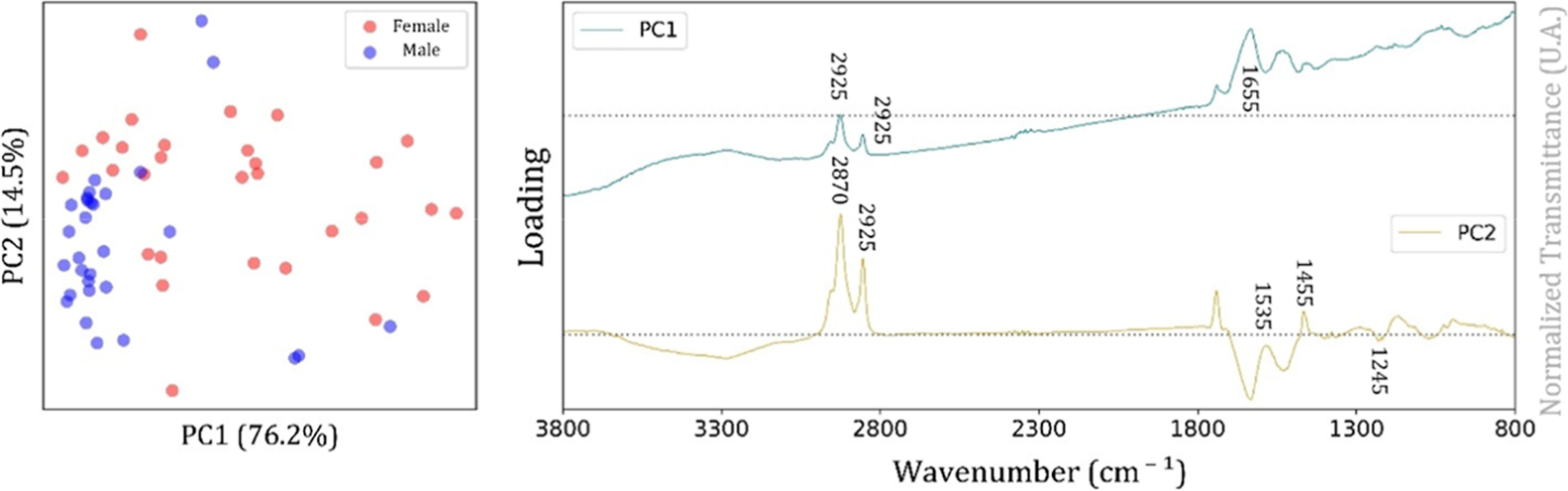
Score plot (left, circles represent sex samples) and loading plot (right, spectral bands of PC1 and PC2) for the Bicudo bird (spectral range from 3800 to 800 cm^–1^).

The two spectral regions mentioned above, together with the full spectral range, were separated into different data sets and used in association with PCA as input data to train the machine-learning models to visualize trends of similarities and/or differences between the bird genders. For the training data, the number of PCs and the number of trees in the Random Forest were optimized to yield the best result in the LOOCV validation. The optimization of the input parameters of the Random Forest for the three spectral regions considered, i.e., the full range (from 3800 to 800 cm^–1^), the ranges from 3000 to 2800 cm^–1^, and from 1800 to 1300 cm^–1^, yielded the best result for the range from 3000 to 2800 cm^–1^, for which a maximum accuracy of 97.6%, with 110 trees and 7 PC’s, was achieved. The trained protocol was then validated using the test data, achieving an accuracy of 94.4%. The corresponding confusion matrices for the best Random Forest classifier configuration of the training (LOOCV) and test are shown, respectively, in Figure 4a,b, where the rows represent the true class and the columns represent the predicted class achieved by the classifier.

**Figure 4:**
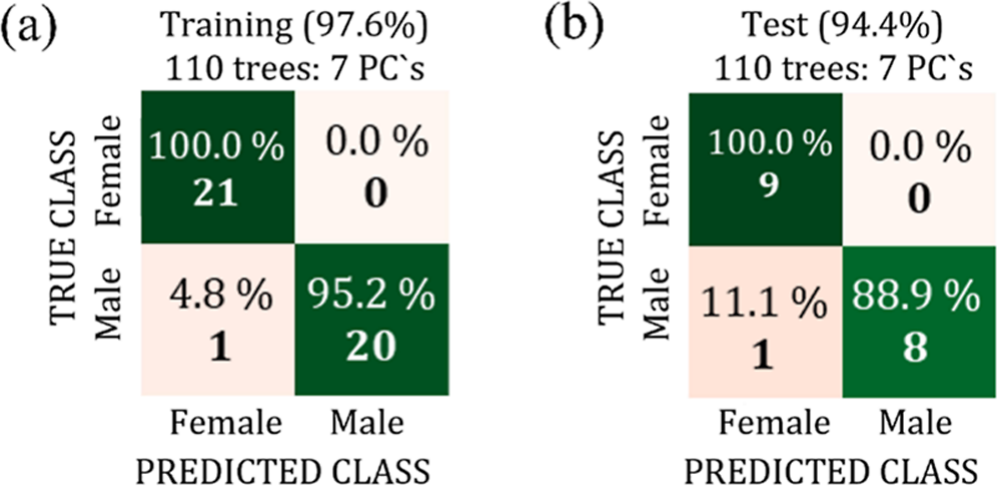
Confusion matrices for the training data set with LOOCV (a) and test data set (b) for the Bicudo bird using the spectral range from 3000 a 2800 cm^–1^.

The very similar values of the test data set accuracy (94.4%) and the training data set accuracy (97.6%) indicated that the model features a good generalization and is unlikely to be suffering from overfitting/underfitting. In particular, the model was able to predict the female class with 100% accuracy, while for the male class, although the result was promising, an accuracy of 88.9% was achieved.

### 3.3 *O. angolensis* (Curio), *N. hollandicus* (Cockatiel), and *P. krameri* (Ring-Necked Parakeet)

A remarkable similarity between the female and male average spectra and standard deviation was shown for the Curio (Figure 5), Cockatiel (Figure 6), and Ring-Necked Parakeet (Figure 7) birds. Some differences can be noted in the highlighted transitions but they are subtle and accompanied by noise, preventing a straightforward univariate analysis.

**Figure 5:**
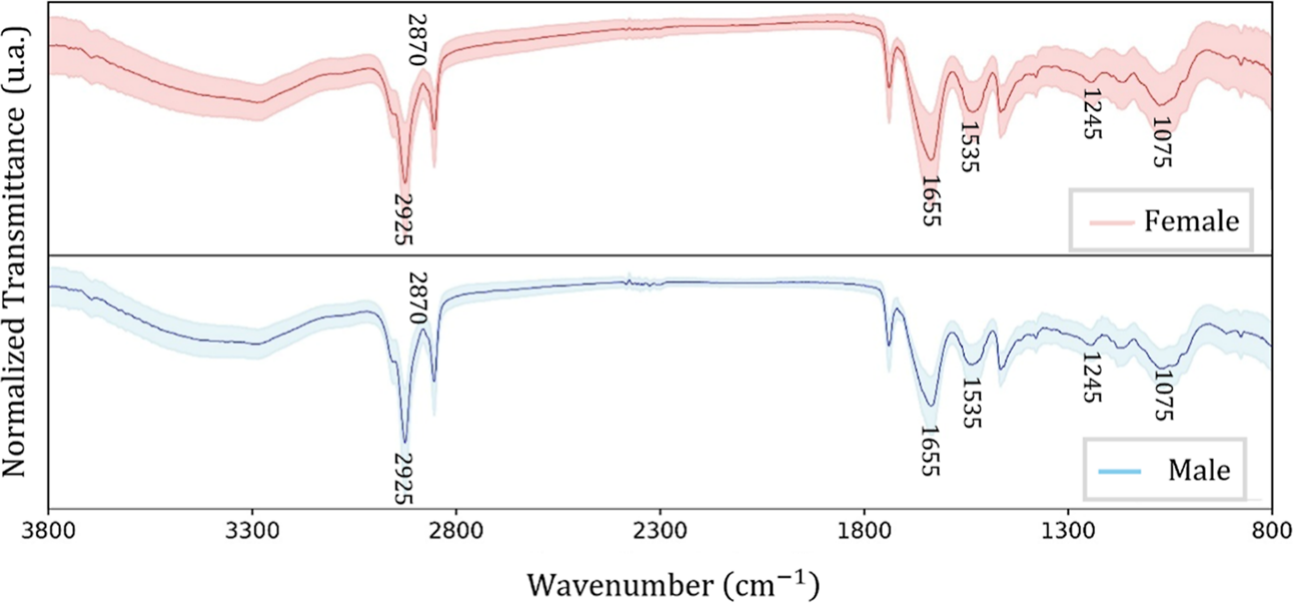
Average FTIR spectra (solid line) and their standard deviation (shaded area) for the Curio bird females (red) and males (blue).

**Figure 6:**
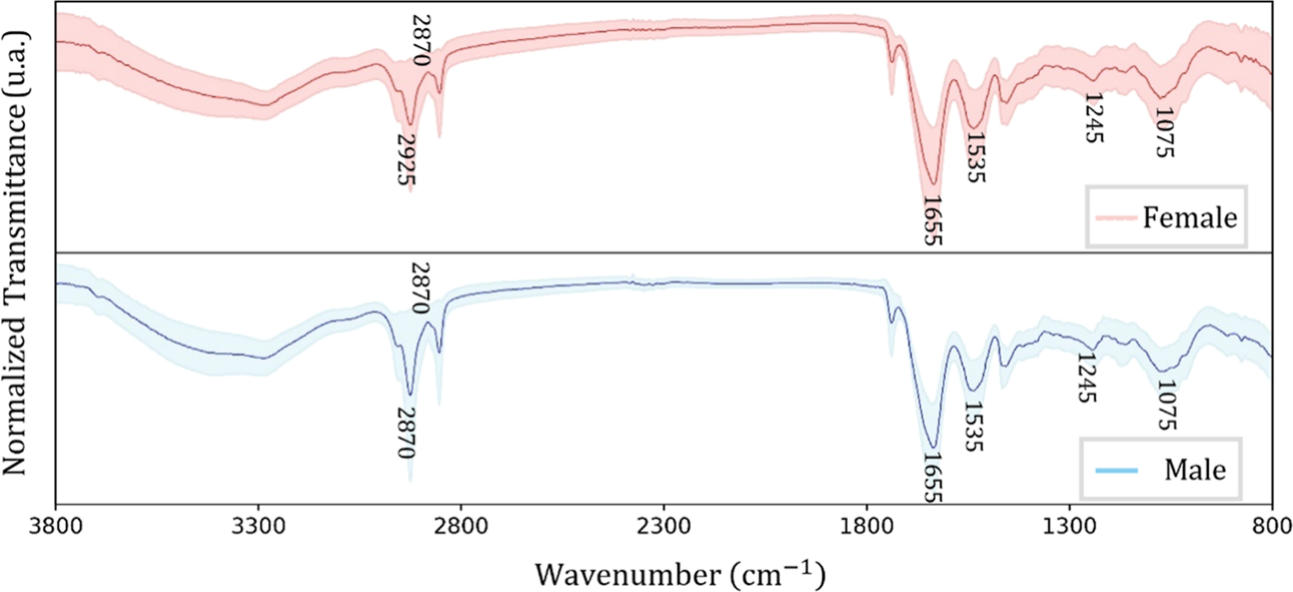
Average FTIR spectra (solid line) and their standard deviation (shaded area) for the Cockatiel bird females (red) and males (blue).

**Figure 7:**
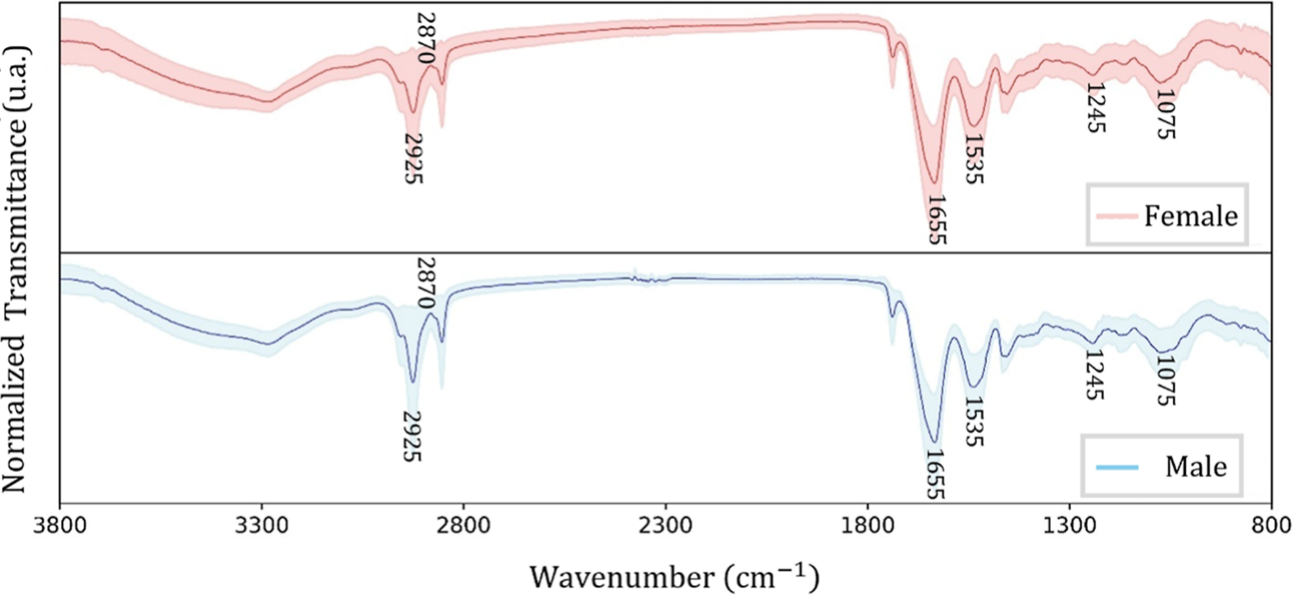
Average FTIR spectra (solid line) and their standard deviation (shaded area) for the Ring-Necked Parakeet bird females (red) and males (blue).

The score plot for PC1 × PC2 and the corresponding loading plot for the spectral region from 3800 to 800 cm^–1^ for the Curio bird, Cockatiel, and Ring Necked birds are shown, respectively, in Figures 8–10. All score plots show the occurrence of a significant variation in the data for the female class compared to the male class, whereas the loading plots indicate a significant contribution of the spectral bands located in the range from 3000 to 2800 cm^–1^ and from 1800 to 1300 cm^–1^. A similar result obtained for Bicudo classification.

**Figure 8:**
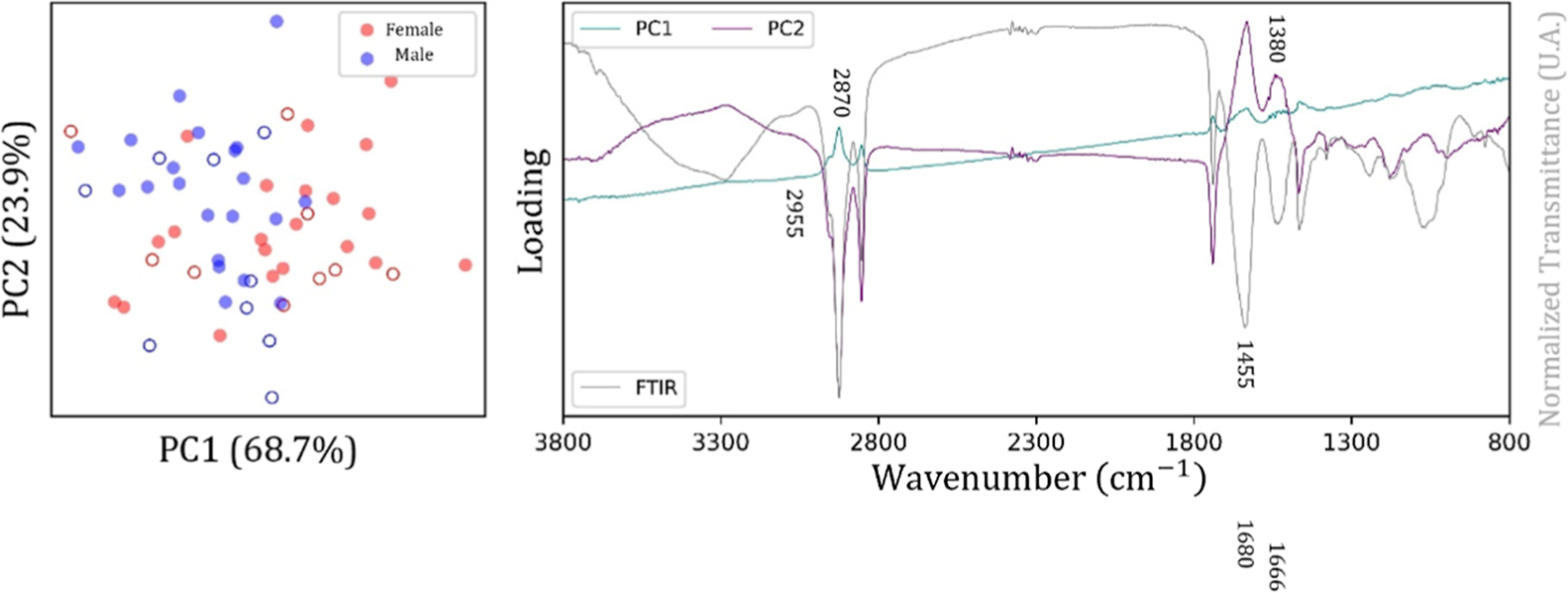
Score plot (left, circles represent samples) and loading plot (right, spectral bands of PC1 and PC2) for the Curio bird (spectral range from 3800 to 800 cm^–1^).

**9:**
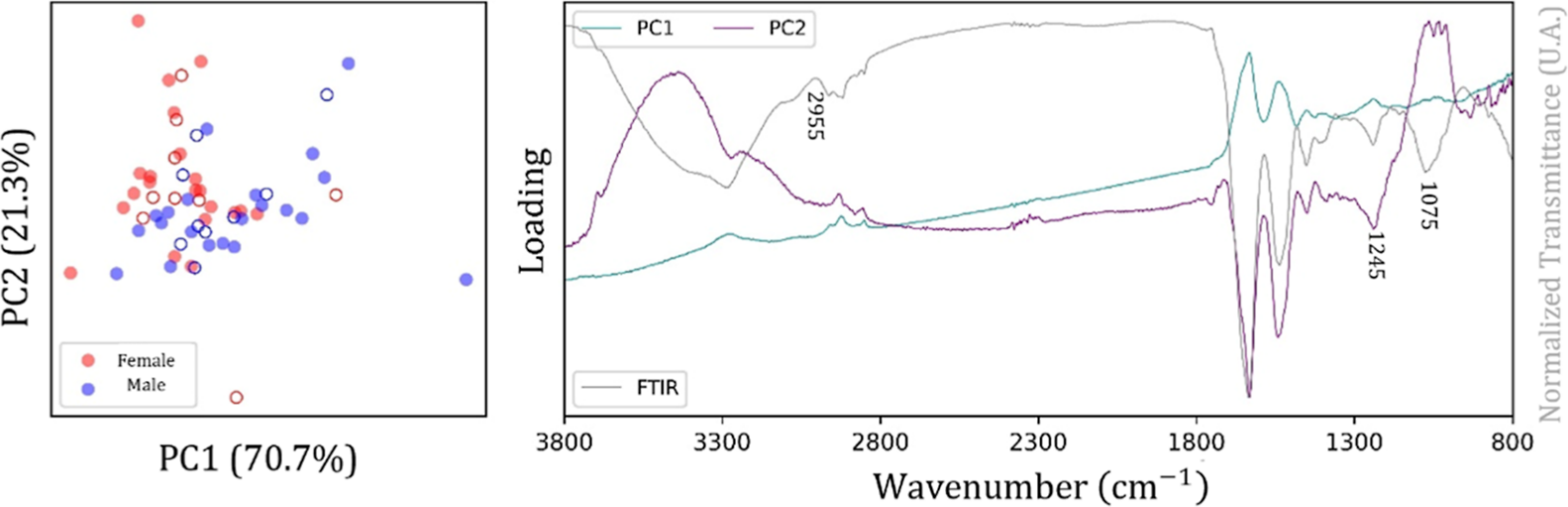
Score plot (left, circles represent samples) and loading plot (right, spectral bands of PC1 and PC2) for the Cockatiel bird (spectral range from 3800 to 800 cm^–1^).

**Figure 10:**
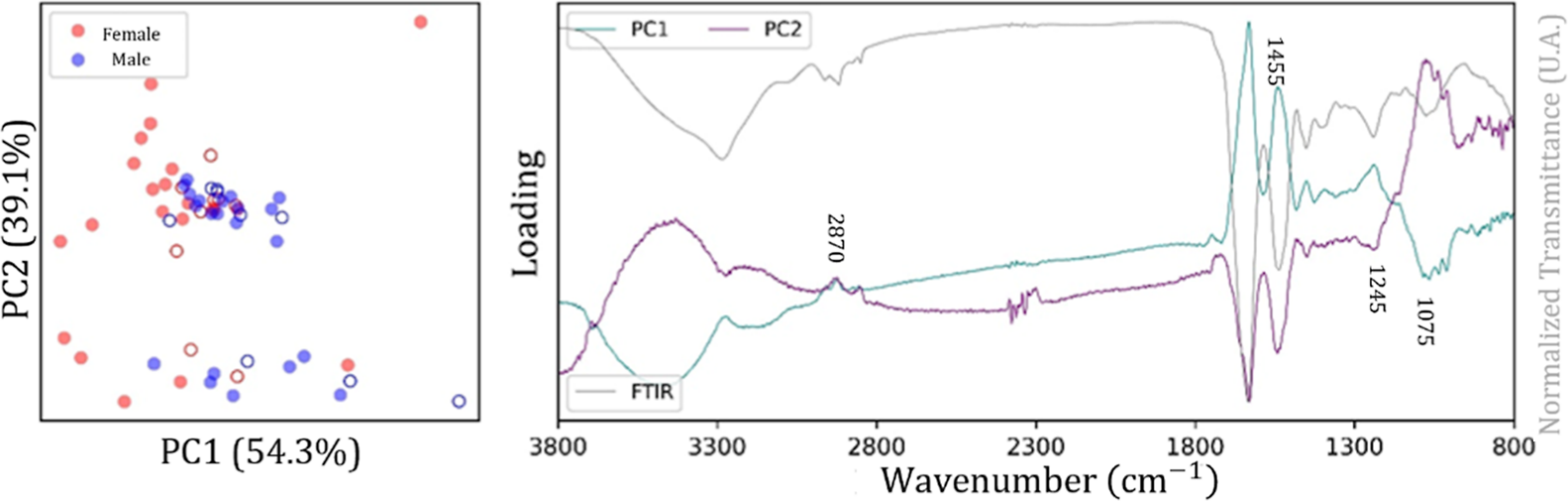
Score plot (left, circles represent samples) and loading plot (right, spectral bands of PC1 and PC2) for the Ring Necked bird (spectral range from 3800 to 800 cm^–1^).

To identify the most promising spectral range capable of differentiating female and male bird genders, the training stage for the machine-learning classifier was performed in the ranges from 3000 to 2800 cm^–1^ and from 1800 to 800 cm^–1^. The overall training accuracies were achieved by the Random Forest classifier using LOOCV during the internal validation step and by optimizing the numbers of trees and PCs used in each training step. The training results showed that the best overall performance was achieved for the range from 3000 to 2800 cm^–1^ for Cockatiel and Ring-necked Parakeet and from 1800 to 800 cm^–1^ for Curio. The training and external validation confusion matrices obtained for the three bird species are shown, respectively, in Figures 11–13.

**Figure 11:**
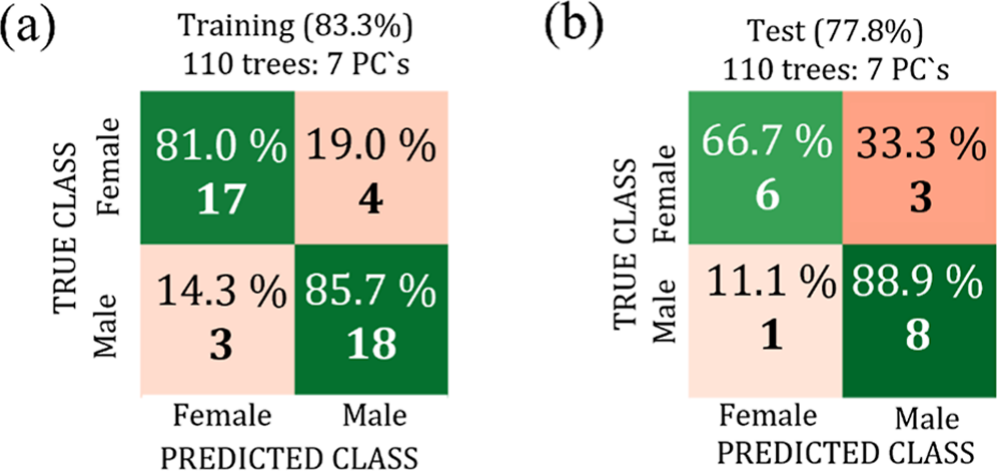
Confusion matrices for the training data set with LOOCV (a) and test data set (b) for the Cockatiel bird using the spectral range from 3000 to 2800 cm^–1^.

**12:**
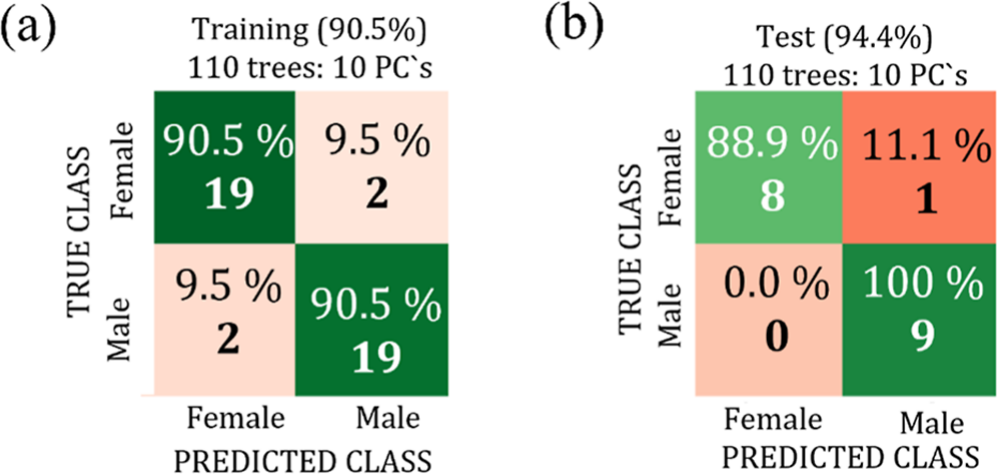
Confusion matrices for the training data set with LOOCV (a) and test data set (b) for the Curio bird using the spectral range from 1800 to 800 cm^–1^.

**Figure 13:**
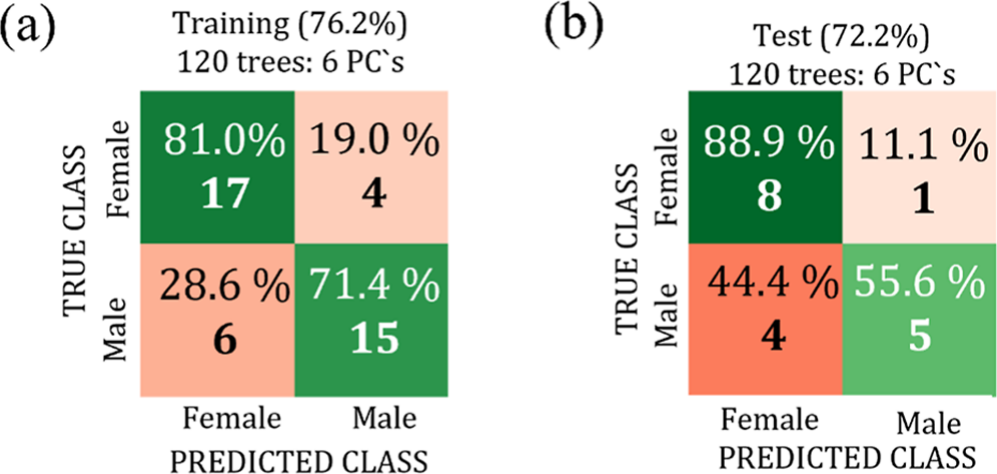
Confusion matrices for the training data set with LOOCV (a) and test data set (b) for the Ring Necked bird using the spectral range from 3000 to 2800 cm^–1^.

### 3.4 Consolidated Results

Table 2 shows a summary of the consolidated results achieved as a function of the analyzed spectral range and bird species, which allows one to compare training and validation accuracies and the number of parameters used in the machine-learning classifier. Satisfactory results with good accuracies for gender determination were obtained for Bicudo (94.4%), Cockatiel (77.8%), and Ring Neck (72.2%) birds in the range for 3000–2800 cm^–1^, while the Curio presented better accuracy (94.4%) for the region of 1800–800 cm^–1^.

**Table 2:** Summarized Results Achieved by Applying the Random Forest Model to Different Spectral Ranges in the Training and Test Stages for the Four Bird Species

bird	interval (cm^–1^)	accuracy (%) training	accuracy (%) test	number of trees	number of PC’s
Bicudo	3800–800	88.1	77.8	100	7
Bicudo	3000–2800	97.6	94.4	110	7
Bicudo	1800–800	97.6	77.8	130	7
Cockatiel	3800–800	78.6	72.2	100	5
Cockatiel	3000–2800	83.3	77.8	110	7
Cockatiel	1800–800	81.0	66.7	190	10
Curio	3800–800	78.6	88.9	140	7
Curio	3000–2800	88.1	66.7	110	10
Curio	1800–800	90.5	94.4	100	10
Ring Neck	3800–800	78.6	66.7	140	4
Ring Neck	3000–2800	76.2	72.2	120	6
Ring Neck	1800–800	76.2	72.2	150	6

A possible explanation for Curio’s better performance in a different region can be related to the noise because both regions present potential for sex gender discrimination. The 3000–2800 cm^–1^ region is dominated by C–H stretching vibrations of aliphatic chains, which are generally less complex and may be influenced by lipid contamination or secretions from the preen gland. While this region provided a satisfactory discrimination for the other species analyzed, in the case of the Curio, the model appears to have overfitted these species-specific particularities, resulting in a high training accuracy (88.1%) but poor generalization in the test set (66.7%). This pattern is a clear indication of overfitting, and thus, the predictive value of this spectral region for Curio should be interpreted with caution. In contrast, the 1800–800 cm^–1^ region comprises a broader and information-rich set of vibrational bands, including amides I, II, and III of keratin, ester C=O, and phosphate vibrations. These features provide stronger biochemical signatures of the feather composition. This is likely to explain the excellent test accuracy observed for Curio in this region (94.4%). These findings reinforce that, although the C–H stretching region may capture useful variability under certain conditions, the amide and carbonyl-rich regions (1800–800 cm^–1^) constitutes a more reliable and biologically meaningful spectral window for sex discrimination in Curio species. To determine whether this finding is exclusive to the Curio species, further studies with a larger sample set are required. Nevertheless, all species demonstrated the potential for sex identification using the FTIR equipment.

## 4 Conclusions

This study highlights the potential application of attenuated total reflectance Fourier transform infrared spectroscopy (ATR-FTIR), combined with principal component analysis (PCA) and Random Forest (RF) algorithms, as a fast, cost-effective, and noninvasive technique for bird gender identification. Analyses were conducted on four bird species: *O. maximiliani* (Bicudo), *O. angolensis* (Curio), *N. hollandicus* (Cockatiel), and *P. krameri* (Ring-necked Parakeet). Excellent results were achieved for *O. maximiliani* and *O. angolensis*, with an external validation accuracy of 94.4%, and promising performances were observed for *N. hollandicus* (77.8%) and *P. krameri* (72.2%). These findings suggest that despite spectral similarities between species and sexes that may limit discrimination, machine learning models trained with optimized spectral regions can effectively distinguish the bird gender in most cases. Nevertheless, further studies are needed to transform this technique into a viable commercial application. The clear discrimination potential observed in this work points to a promising path toward the development of a more robust protocol for spectroscopic-based gender identification using simple and noninvasive measurements. The results support ATR-FTIR spectroscopy as a strong candidate to complement or even substitute for molecular DNA methods for bird gender identification. Expanding this research with larger data sets, various feather types, and alternative machine learning strategies may enhance the reliability and scalability of the proposed approach.
